# Large polarization gradients and temperature-stable responses in compositionally-graded ferroelectrics

**DOI:** 10.1038/ncomms14961

**Published:** 2017-05-10

**Authors:** Anoop R. Damodaran, Shishir Pandya, Yubo Qi, Shang-Lin Hsu, Shi Liu, Christopher Nelson, Arvind Dasgupta, Peter Ercius, Colin Ophus, Liv R. Dedon, Josh C. Agar, Hongling Lu, Jialan Zhang, Andrew M. Minor, Andrew M. Rappe, Lane W. Martin

**Affiliations:** 1 Department of Materials Science and Engineering, University of California, Berkeley, California 94720, USA; 2 Department of Chemistry, The Makineni Theoretical Laboratories, University of Pennsylvania, Philadelphia, Pennsylvania 19104-6323, USA; 3 Geophysical Laboratory, Carnegie Institution for Science, Washington, District of Columbia 20015, USA; 4 National Center for Electron Microscopy, The Molecular Foundry, Lawrence Berkeley National Laboratory, Berkeley, California 94720, USA; 5 Department of Physics and Astronomy, Rutgers University, Piscatway, New Jersey 08854, USA; 6 Materials Science Division, Lawrence Berkeley National Laboratory, Berkeley, California 94720, USA

## Abstract

A range of modern applications require large and tunable dielectric, piezoelectric or pyroelectric response of ferroelectrics. Such effects are intimately connected to the nature of polarization and how it responds to externally applied stimuli. Ferroelectric susceptibilities are, in general, strongly temperature dependent, diminishing rapidly as one transitions away from the ferroelectric phase transition (*T*_C_). In turn, researchers seek new routes to manipulate polarization to simultaneously enhance susceptibilities and broaden operational temperature ranges. Here, we demonstrate such a capability by creating composition and strain gradients in Ba_1−*x*_Sr_*x*_TiO_3_ films which result in spatial polarization gradients as large as 35 μC cm^−2^ across a 150 nm thick film. These polarization gradients allow for large dielectric permittivity with low loss (*ɛ*_r_≈775, tan *δ*<0.05), negligible temperature-dependence (13% deviation over 500 °C) and high-dielectric tunability (greater than 70% across a 300 °C range). The role of space charges in stabilizing polarization gradients is also discussed.

## Abstract (web summary)

Future technologies based on ferroelectric materials will require new routes to control the nature of polar order. Through a combined experimental and theoretical study of compositionally and strain-graded Ba1-xSrxTiO3 heterostructures, Damodaran *et al*. demonstrate the ability to engineer large polarization gradients and temperature-stable susceptibilities.

There has been continued interest in developing routes to control the nature of polar order in ferroelectric materials so as to maximize a desired susceptibility over a wide range of temperatures^1^,^2^,^3^,^4^. One important application driving such research involves the development of high-performance polar materials that combine high dielectric response and electric-field tunability, low dielectric loss, and excellent temperature stability (<5% change over a 500 °C range); a combination which has been touted as one of the most significant materials-related challenges in the integration of frequency-agile, wireless devices in wide-band-gap microelectronics^5^,^6^,^7^,^8^. Traditionally, control of polar order for temperature-stable responses in bulk materials has been accomplished via chemical routes whereby symmetry and transition temperatures can be tuned by chemical alloying. Examples of this approach include the use of chemistry to create morphotropic phase boundaries^9^ (a temperature-insensitive structural phase boundary) where competing ferroelectric phases interact with external fields to yield high susceptibilities, and relaxor ferroelectrics where compositionally-induced disorder and related random fields result in a glass-like state of nanopolar regions that exhibit broad temperature dependence and enhanced frequency-dependent dielectric and other functional responses^10^. Losses associated with the large piezoelectric effects in such systems, however, hinder their use in tunable applications^11^,^12^. Another route to control polar order for temperature-stable responses includes the use of improper ferroelectrics, where the electric polarization is not the primary order parameter, but is coupled to another primary structural order parameter^13^. While this yields a dielectric permittivity that does not obey classical Curie–Weiss behaviour and can be essentially constant with temperature, it also results in a similarly weak electric-field dependence and low polarization-related susceptibilities^13^. Ultimately, the general design consideration has been that the higher the dielectric constant, the higher the tunability, loss, and temperature dependence of the dielectric permittivity; something which has remained true for most material systems^14^. Therefore, novel pathways to decouple such interdependencies are in critical need to enable next-generation functionalities.

With the advent of modern thin-film deposition approaches, new ways to control polarization have begun to emerge. Epitaxial strain, in particular, provides an important route by which one can control the structure, polarization and properties of ferroelectrics^15^,^16^. There has been little work, however, on the simultaneous combination of chemistry and strain as a way to control polarization. One way researchers have attempted to achieve this combined control is by creating compositionally- graded heterostructures. Extensive theoretical treatments of such structures have predicted a range of interesting effects including the potential for large and temperature-stable ferroelectric susceptibilities^17^,^18^,^19^,^20^. Early experimental work, mostly on Ba_1–*x*_Sr_*x*_TiO_3_-based ferroelectrics, primarily focused on relaxed films (that is, not coherently strained to the substrate) wherein only internal strains between layers are present. Nonetheless, this work demonstrated the potential to stabilize a more diffuse ferroelectric-to-paraelectric phase transition generating less temperature-dependent response^21^,^22^,^23^. More recently, researchers have been able to produce coherently strained and compositionally- graded ferroelectric heterostructures^24^,^25^,^26^,^27^. Despite this interest, the true nature of polarization and strain gradients in these heterostructures remains unstudied. Furthermore, the question of how polarization should evolve in these heterostructures remains unanswered since several competing factors including depolarization^28^, flexoelectric effects^29^, and the presence of charged defects^30^ are all critical in understanding how the polarization will evolve spatially and with temperature^20^.

In this work, we directly measure and understand the nature of polarization evolution in compositionally- and strain-graded heterostructures, and provide a potential route for on-demand optimization of polarization profiles that can overcome limitations and coupled responses in uniform composition materials. In particular, using a combination of thin-film synthesis, X-ray reciprocal space mapping (RSM) studies, scanning transmission electron microscopy (STEM)-based mapping of strain and polar atomic displacements at the nanoscale, ferroelectric and dielectric measurements, and first-principles-based molecular dynamics (MD) simulations we observe and understand the stabilization of a polarization gradient as large as 35 μC cm^−2^ per 150 nm in compositionally-graded Ba_1−*x*_Sr_*x*_TiO_3_ heterostructures. Such heterostructures also exhibit a large average dielectric permittivity (*ɛ*_r_≈775) which deviates <13% over a 500 °C temperature range, dielectric tunability >70% across a 300 °C temperature window above room temperature, and low loss (tan *δ*<0.05) behaviour. The gradient in polarization is likely stabilized by space charges which, in turn, work to reduce electrical losses over a wide temperature range.

## Results

### Design of Ba_1−*x*_Sr_*x*_TiO_3_-based thin-film heterostructures

**Figure 1:**
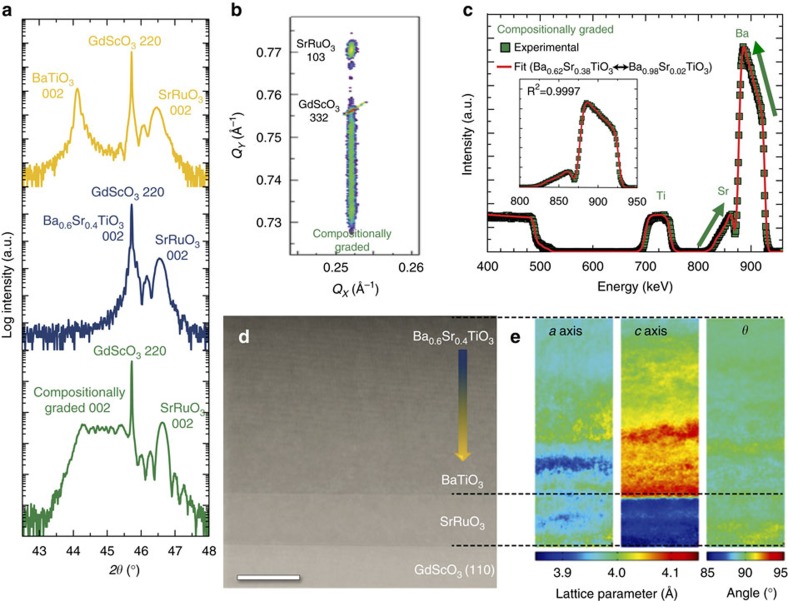
haracterization of composition- and strain- gradients in Ba_1−*x*_Sr_*x*_TiO_3_ thin film heterostructures synthesized. (**a**) XRD studies about the 002-diffraction condition for (top to bottom) BaTiO_3_, Ba_0.6_Sr_0.4_TiO_3_, and compositionally graded heterostructures. (**b**) Off-axis RSM studies about the pseudocubic 103- and 332-diffraction conditions of a compositionally-graded heterostructure and the GdScO_3_ substrate, respectively, showing that the film is coherently strained to the substrate. (**c**) Rutherford backscattering spectrum for a compositionally graded heterostructure revealing a strong compositional gradient and the corresponding best fit for the data. (**d**) Low-resolution STEM image of a compositionally-graded heterostructure revealing no obvious defects within the films and pristine interfaces between layers. Scale bar, 50 nm. (**e**) Corresponding 2D maps of (left to right) in-plane (*a* axis) and out-of-plane (*c* axis) lattice parameters, and shear distortions (*θ*) of the compositionally-graded film extracted from local nanobeam diffraction studies.

We have examined three different variants of 150 nm thick Ba_1−*x*_Sr_*x*_TiO_3_-based heterostructures grown on SrRuO_3_-buffered GdScO_3_ (110) substrates: (1) single-layer BaTiO_3_, (2) single-layer Ba_0.6_Sr_0.4_TiO_3_, and (3) compositionally-graded films that transition linearly from BaTiO_3_ to Ba_0.6_Sr_0.4_TiO_3_ from substrate to surface (compositionally-graded heterostructures; Methods and Supplementary Fig. 1). Rationale for the selection and design of these heterostructures is expanded upon in the Supplementary Information. Wide-angle X-ray diffraction (XRD) *θ*–2*θ* scans reveal that all heterostructures are epitaxial, fully (001)-oriented, and single phase (Methods and Supplementary Fig. 2). A zoom-in of the X-ray line scans about the 002-diffraction condition (Fig. 1a) reveals well-defined crystalline film peaks for the uniform-composition BaTiO_3_ and Ba_0.6_Sr_0.4_TiO_3_ heterostructures while the compositionally-graded heterostructures exhibit a plateau in the diffraction intensity that stretches between the peak positions of the uniform-composition end-members. Off-axis X-ray RSM studies about the 103- and 332-diffraction conditions of the compositionally-graded heterostructure and substrate, respectively, reveal the in-plane epitaxy and that the films remain coherently strained to the substrate (Fig. 1b). The film *c-*axis lattice parameter stretches across values corresponding to the strained end-member compositions (from 3.965 Å for Ba_0.6_Sr_0.4_TiO_3_ to 4.075 Å for BaTiO_3_). A compositional gradient from Ba_0.62_Sr_0.38_TiO_3_ to Ba_0.98_Sr_0.02_TiO_3_ from surface to substrate interface is confirmed by Rutherford backscattering spectrometry (RBS; Methods and Fig. 1c). We note that these compositional gradients are found to be very stable even after high-temperature anneals (Supplementary Fig. 3). High-angle annular dark-field STEM (HAADF-STEM) *Z*-contrast imaging (Methods) of the compositionally-graded heterostructures (Fig. 1d) reveals the presence of sharp interfaces without evidence of misfit dislocations or other extended defects (Supplementary Fig. 4). STEM-based nano-beam electron diffraction measurements (Methods and Supplementary Fig. 5)^31^ provide localized strain maps across the film thickness with nanometer-scale resolution. The colour-coded two dimensional (2D) maps for lattice parameter evolution (Fig. 1e) show nearly uniform colour contrast for the in-plane *a*-axis lattice parameter and shear distortions (*θ*), while the out-of-plane *c*-axis lattice parameter is seen to vary continuously by nearly 2.7% across the compositionally-graded heterostructure; confirming the presence of large in-plane and out-of-plane strain gradients of −6.2 × 10^4^ m^−1^ and 1.2 × 10^5^ m^−1^, respectively, relative to the thickness-dependent bulk, pseudocubic lattice parameters of the heterostructure.

### Probing polarization evolution in compositionally-graded heterostructures

**Figure 2:**
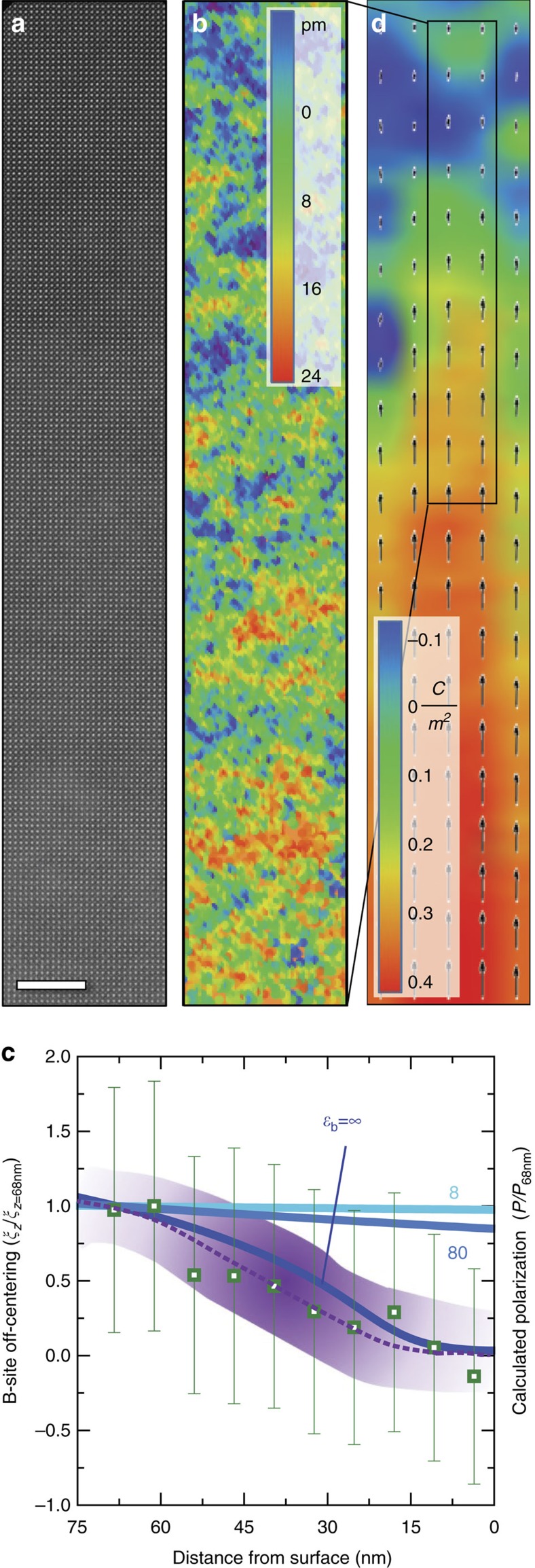
Polarization gradients in compositionally-graded Ba_1−*x*_Sr_*x*_TiO_3_ heterostructures. (**a**) Cross-section HAADF-STEM image of the top 72 nm of a compositionally-graded heterostructure. Scale bar, 5 nm. (**b**) 2D map of local displacement of the Ti ion showing a smooth gradient in the displacement (and therefore polarization) as a function of position. (**c**) Experimentally extracted normalized values for the out-of-plane off-centring of the Ti cation (left axis, green data obtained by binning data from the HAADF-STEM image into 7.2 nm (tall) × 12 nm (wide) regions along the *z*-direction with error bars indicative of the s.d. of the data) together with the polarization variation predicted by the molecular dynamics simulations (purple dashed line, with shaded area showing the spread of the simulations) and GLD models (blue curves, right axis) for various background permittivity values (*ɛ*_b_=8, 80 and ∞). (**d**)Molecular dynamics simulation of a compositionally-graded heterostructure showing a large gradient in the polarization throughout the thickness of the heterostructure.

Recent Ginzburg–Landau–Devonshire (GLD)-based phenomenological models for such strain- and compositionally-graded heterostructures^20^ have shown that the relative strength of the depolarization effects can strongly influence the polarization profile across the film thickness. These effects are predicted to be strongest at compositions corresponding to the Sr-rich portion of the heterostructures where depolarization fields can induce polar order in the otherwise non-polar material. To test this expectation, we probed the local polarization in the film using HAADF-STEM-based polarization mapping, focusing on the top 72 nm (the Sr-rich region) of the compositionally-graded heterostructures (Methods, Fig. 2a). From the data, a 2D map of the out-of-plane displacement (*ξ*_z_) of the central Ti-ion was extracted at the unit-cell level (Fig. 2b). This analysis reveals the presence of a large gradient in the polarization across the film thickness, as well as a preferred direction of polarization that points from the bottom to the top of the film. To provide a statistically meaningful data set from the HAADF-STEM imaging, we have binned the data into 7.2 nm (tall) × 12 nm (wide) regions and extracted an average displacement vector (with error bars indicative of the standard deviation of the data, green squares, Fig. 2c). These data suggest that the polarization is essentially zero at the surface of the compositionally-graded heterostructure and subsequently increases to an average value of 25 μC cm^−2^ (computed from an average Ti-ion displacement of 12 pm)^32^ upon transitioning 72 nm into the film. Such a polarization profile is contrary to what is predicted by the GLD models^20^, which predict nearly uniform polarization for standard values of background dielectric constant (*ɛ*_b_, a parameter that determines the magnitude of depolarization effects from spatial gradients in ferroelectric polarization)^33^ of 8 and 80 (blue curves, Fig. 2c). Large variations in the polarization across the thickness, consistent to those observed here experimentally, are only achieved when *ɛ*_b_→∞. Such a value could imply that the Ba_1−*x*_Sr_*x*_TiO_3_ heterostructure behaves as a metal; however, this is clearly not the case as experiments show that it is a rather good insulator (Supplementary Fig. 6). Instead, what the need for the infinite value of the background dielectric constant reveals is that, in this heterostructure, depolarization effects are essentially turned off. The fact that large polarization gradients (a change of 25 μC cm^−2^ across the 72 nm region imaged here) exist in these compositionally-graded heterostructures hints at the presence of some mechanism that screens the divergence in polarization.

To understand the stabilization of such large polarization gradients at the microscopic level, we employed MD simulations for a compositionally-graded heterostructure (Methods). Akin to the experimental data, a 2D colour map of the polarization extracted from a section of the MD simulation (Fig. 2d) reveals that a large polarization gradient (a change of 35 μC cm^−2^ across the entire thickness of the film) is preferred over a uniform polarization state and that the trend in the polarization (purple dashed and region, Fig. 2c) matches well with the experimentally extracted polarization profile. Further investigation of the MD simulation (Supplementary Fig. 7) reveals a tendency for the system to create 180° domain structures as a mechanism for screening depolarization effects that arise from the polarization gradient within the compositionally-graded heterostructures^34^. Extensive piezoresponse force microscopy studies (Supplementary Fig. 8), however, reveal uniform contrast in both the vertical and lateral piezoresponse signals; indicative of the films being monodomain. Thus, while, both the MD simulations and experiments confirm large gradients in the magnitude of the polarization across the film thickness as the stable ground state, the mechanism they adopt to screen depolarization effects arising from such gradients are different. In the absence of 180° domains, a non-zero spatial divergence in ferroelectric polarization across the film thickness is only possible in the presence of screening charges (since ∇·*P*=*σ*_b_). For a change of polarization of 35 μC cm^−2^ over 150 nm, an average charge density of 1.5 × 10^19^ cm^−3^ is required. This screening charge need not only be made up of mobile carriers (namely electrons and/or holes), but can be some combination of mobile electrons or holes, charged defects, and so on, within the film. For reference, to produce the charge density noted here, one needs only have non-stoichiometry on the order of 0.05 atomic percent; in other words, deviation of the Ba, Sr, or Ti content from ideal stoichiometry by only 0.05 atomic percent could give rise to enough defects to compensate this polarization gradient (even in the absence of mobile electrons/holes). Consistent with this, it is commonly known that oxide ferroelectrics can possess a high density of charged defects, with the best single crystals possessing defect densities of 10^17^ cm^−3^ (refs 35, 36) and thin-film versions possessing densities as high as 10^19^ cm^−3^ (ref. 37). More specifically, based on the preferred direction of the polarization and the large polarization gradients observed experimentally, a distribution of negative space charges would be required to screen the polarization gradients (Supplementary Fig. 9). It is known that Ba_*x*_Sr_1−*x*_TiO_3_-based films grown under oxidizing conditions are typically p-type semiconductors^38^, and for such systems negative screening charges can be generated via a depletion of the majority carriers that leave behind an electrically-charged depletion region of immobile/fixed negatively-charged defects. As an added benefit, such a depletion of majority carriers is seemingly beneficial as it also gives rise to a reduction in the leakage currents (Supplementary Fig. 6).

Such a preferred direction of polarization and depletion of majority carriers, in turn, gives rise to a built-in field and can be probed as a shift in the ferroelectric hysteresis loop along the electric-field axis^39^. At the same time, the presence of large strain gradients can also result in built-in fields from flexoelectric effects intrinsic to strain- and compositionally-graded heterostructures^40^,^41^. Polarization-electric field hysteresis loops for the uniform-composition and compositionally-graded heterostructures (Methods) reveal that uniform-composition heterostructures exhibit centred loops, while as-grown compositionally-graded heterostructures exhibit a positive offset of 25 kV cm^−1^ (Supplementary Fig. 10a); confirming the preferred upward polarization direction for the as-grown compositionally-graded heterostructures. To understand the relative importance of the extrinsic space charge and intrinsic flexoelectric driving forces for such shifted loops, we conducted field-cooling studies on the compositionally-graded heterostructures. Samples were heated to 500 °C and subsequently cooled under applied electric fields. Cooling under negative fields (which further reinforces the as-grown polarization direction) results in no change in the nature of the hysteresis loop or built-in potential, while cooling under positive fields (which switches the polarization from the as-grown state) results in reversal of the built-in potential (−30 kV cm^−1^, Supplementary Fig. 10b). This field dependence of the loop shift is reversible and repeatable. Since the direction of the strain and composition gradient, and the sign of the flexoelectric coefficient, are all invariant in these experiments, the resulting reversal of the built-in potential indicates that the built-in fields arise primarily from extrinsic space charges that screen polarization gradients and that the flexoelectric contribution is minimal. To summarize thus far, we have directly measured the presence of a large spatial gradient in ferroelectric polarization in compositionally-graded Ba_1−*x*_Sr_*x*_TiO_3_ heterostructures and highlighted the importance of screening space charges (which are ubiquitous in real materials) in stabilizing the polarization gradient and subsequent built-in potential in these materials.

### Thermal stability of dielectric responses

**Figure 3:**
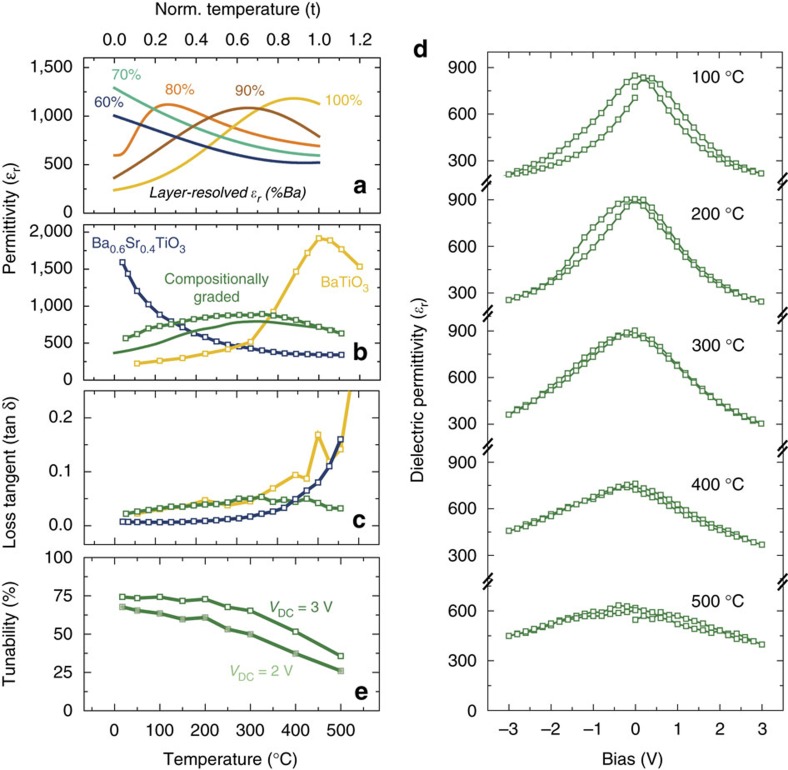
Wide-range temperature-stable dielectric permittivity. (**a**) Layer-resolved (labelled by the percentage of Ba in the given layer) permittivity versus normalized temperature 
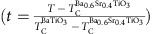
 curves from the simulations. (**b**) Dielectric permittivity as extracted from experiments (open squares) for single-layer BaTiO_3_ (yellow) and Ba_0.6_Sr_0.4_TiO_3_ (blue) heterostructures as well as a compositionally-graded heterostructures (green) along with simulations (solid line) for compositionally-graded (green) heterostructure. (**c**) loss tangent for single-layer BaTiO_3_ (yellow) and Ba_0.6_Sr_0.4_TiO_3_ (blue) heterostructures as well as a compositionally-graded heterostructures (green). (**d**) Temperature-dependence of the dielectric permittivity as a function of applied bias for the compositionally-graded heterostructure from 100 to 500 °C. (**e**) Extracted tunability 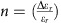 for the compositionally-graded heterostructure as a function of temperature.

Having established the complex spatial evolution of polarization in a compositionally- graded heterostructure, we now focus on its implications for the temperature and electric-field dependence of the ferroelectric susceptibilities. We begin by exploring MD simulations (Methods) of the temperature-dependent relative dielectric permittivity *ɛ*_r_ which can be calculated using


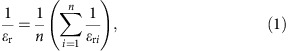


where *ɛ*_r*i*_ is the relative dielectric permittivity of the *i*th virtual uniform composition layer, as calculated by the Kubo formula, in which the permittivity is proportional to the square of the local polarization fluctuations. First, the permittivity for various uniform-composition layers within the compositionally-graded heterostructures are explored for comparison (marked by the Ba content in the different layers, Fig. 3a). As expected, the dielectric response of these individual layers is found to be strongly temperature dependent; peaking near the *T*_C_ predicted for their specific chemical composition and strain state. The computed overall permittivity of the compositionally-graded heterostructures, on the other hand, shows a broad and relatively flat temperature dependence that is dramatically different from the uniform-composition layers (solid green line, Fig. 3b). This is explained by the fact that, at any temperature, the overall permittivity is the harmonic mean of layers with positive and negative temperature coefficients of permittivity (equation 1) and is therefore biased towards the temperature-stable, lower permittivity layers.

These predictions are easily verified by direct measurement of the temperature-dependent, out-of-plane dielectric permittivity of the uniform-composition and the compositionally-graded heterostructures. Dielectric measurements on uniform-composition BaTiO_3_ and Ba_0.6_Sr_0.4_TiO_3_ heterostructures reveal strong temperature-dependent response in the vicinity of their respective *T*_C_ (blue and yellow data for Ba_0.6_Sr_0.4_TiO_3_ and BaTiO_3_, respectively, Fig. 3b). The compositionally-graded heterostructures, on the other hand, demonstrate remarkably different trends and possess consistently high values of dielectric permittivity (average *ɛ*_r_≈775) with a deviation of <13% over the entire temperature range from 25 to 500 °C (green data, Fig. 3b); matching well with the MD simulations. The measurements also reveal diminished dielectric loss (especially at elevated temperatures) in the compositionally-graded heterostructures (tan *δ*<0.05 from 25 to 500 °C, green data, Fig. 3c) which can be attributed to diminished contributions from the space charges that act as bound charges to screen the non-zero divergence in polarization. Furthermore, the compositionally-graded heterostructures reveal strong electric-field dependence of permittivity across the entire temperature range measured (Fig. 3d). A summary of the tunability at CMOS-compatible voltages (for example, 2 V (133 kV cm^−1^) and 3 V (200 kV cm^−1^)), reveals that the compositionally-graded heterostructures exhibit dielectric tunability exceeding 70% from 25 to 300 °C (Fig. 3e). Thus, compositionally-graded heterostructures allow for a simultaneous optimization of dielectric responses, tunability, dielectric losses, and thermal stability; a feat not easily achieved in uniform-composition films. Such wide-range temperature stability, large response, and strong external-field control are a consequence of the ferroelectric phase transition that sweeps across the compositionally-graded film thickness as the temperature is varied, and can therefore likely be extended to other ferroelectric susceptibilities as well.

## Discussion

Using a combined experimental and theoretical study of model epitaxial, single-crystal, monodomain, and compositionally- and strain-graded Ba_1–*x*_Sr_*x*_TiO_3_ heterostructures, it has been demonstrated that one can engineer large polarization gradients and desirable temperature-stable susceptibilities. The depolarization effects that may arise from such a gradient in polarization are, in a real film, screened by the appropriate distribution space charges that are ubiquitous in these oxide materials. In turn, the magnitude of polarization at any layer is determined by the composition and strain alone and the preferred direction of polarization in the as-grown state is set by the type of defects and the semiconducting nature of the system. Electric-field control of the shifts of the ferroelectric hysteresis loops, as well as observations from the MD simulations, suggest that flexoelectric effects play a minimal role in setting the preferred polarization direction. Thus, these studies have demonstrated that using composition and strain gradients in ferroelectrics, one can deterministically control polarization to produce exotic polarization profiles. In turn, such polarization profiles can induce unprecedented ferroelectric susceptibilities including large, temperature-stable and low-loss dielectric permittivity. Ultimately, our ability to tune the nature of polarization in materials enables us to produce responses more akin to (or even better than) those observed in materials with temperature-insensitive responses (for example, morphotropic phase boundaries, relaxors), and represents a major milestone towards developing ferroelectric-based functionalities for next-generation applications.

## Methods

### Heterostructure synthesis using pulsed-laser deposition

The various heterostructures were grown on GdScO_3_ (110) substrates which were affixed to the heater using Ag-paint and subsequently heated to the deposition temperatures in a dynamic oxygen pressure of 100 mTorr. Growth of the 40 nm SrRuO_3_ bottom electrode was completed from a SrRuO_3_ target (Praxair) at a heater temperature of 635 °C, in a dynamic oxygen pressure of 100 mTorr, at a laser fluence of 1.5 J cm^−2^ and a laser repetition rate of 14 Hz. The various 150 nm thick Ba_*x*_Sr_1−*x*_TiO_3_ films were subsequently grown from two uniform-composition BaTiO_3_ and Ba_0.6_Sr_0.4_TiO_3_ targets (Praxair) at a heater temperature of 600 °C, in a dynamic oxygen pressure of 20 mTorr, at a laser fluence of 1.5 J cm^−2^ and a laser repetition rate of 2 Hz. The compositionally-graded heterostructures were synthesized using a programmable target rotator (Neocera, LLC) that was synced with the excimer laser to controllably vary the number of laser pulses on the BaTiO_3_ and Ba_0.6_Sr_0.4_TiO_3_ targets to achieve the desired composition gradient. Following growth, all heterostructures were cooled to room temperature in a static oxygen pressure of 760 Torr at 5 °C min^−1^.

### X-ray diffraction studies

Wide-angle *θ*−2*θ* XRD patterns and reciprocal space maps were obtained with a Panalytical X'Pert Pro XRD machine with a Cu source.

### Chemical analysis *via* Rutherford backscattering spectrometry

RBS was employed to confirm the generation of compositionally-graded heterostructures. The spectra were taken with a 3.04 MeV He ion beam incident at an angle of 22.5° relative to the sample normal. The backscattered He ions were collected by a silicon surface-barrier detector positioned at 168° with respect to the incident beam. The SIMNRA software package was used to simulate the RBS spectra and obtain the composition of the film. *R*^2^ analysis was performed about the Ba, Sr and Ti peaks to avoid artificially increasing the value of *R*^2^ with the inclusion of substrate peaks. *R*^2^ was calculated using 
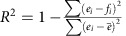
, where *e*- and *f*-values correspond to experimental and simulated data, respectively. RBS studies on the compositionally graded heterostructures reveal a film that smoothly transitions from Ba_0.62_Sr_0.38_TiO_3_ to Ba_0.98_Sr_0.02_TiO_3_ from surface to substrate interface (Fig. 1c) with excellent values of fitting (*R*^2^>0.999).

### High-resolution STEM

Cross-sectional STEM samples were prepared by mechanical polishing on an Allied High-Tech Multiprep. Samples were subsequently Ar-ion milled using a Gatan Precision Ion Milling System with starting energies of 4 keV stepped to a final cleaning energy of 200 keV. High-resolution, HAADF-STEM imaging was performed on the TEAM 0.5 microscope, an aberration-corrected FEI Titan 80–300 operated at 300 keV with a beam current of 60 pA and convergence semi-angle of 17.1 mrad, at the National Center for Electron Microscopy, Lawrence Berkeley National Laboratory.

### Nano-beam electron diffraction

Elastic strain and local crystal rotation mapping was conducted using nano-beam electron diffraction in the TEAM I microscope, an FEI Titan 80–300 operated at 300 kV using a K2 IS direct electron detector, at the National Center for Electron Microscopy, Lawrence Berkeley National Laboratory^31^.

### Extracting polar displacement from high-resolution STEM

The local non-centrosymmetry of the cation positions in the high-resolution (HR)-STEM images was probed as a measure of the local polarization. Ba_*x*_Sr_1−*x*_TiO_3_ adopts a tetragonal structure wherein the oxygen anions and Ti cations shift along the polarization axis relative to the Ba/Sr sublattice to create the electrical dipole. The direction and magnitude of this polarization is inferred using the cations^42^, Ba/Sr and Ti, which have strong signal-to-noise in Z-contrast HR-STEM images.

The polar displacements are measured from Cs aberration corrected HAADF-STEM Z-contrast images, as described above, which have undergone additional spatial distortion correction. A pair of images was acquired from an identical region of the compositionally graded Ba_*x*_Sr_1−*x*_TiO_3_ film using orthogonal scan axes. These were input into a multi-image raster distortion correction algorithm which iteratively determines best fit scan-line level affine transformations to enforce self-consistency between the images^43^.

Displacement vectors corresponding to local offsets between the A- and B-site sublattices were calculated by first determining atomic positions from a fit to 4-parameter spherical Gaussians using a trust-region algorithm in Matlab. Fits were performed simultaneously for 25-atom clusters centred on the A-sites. For each local fit the positions of the inner nine atoms of the cluster were saved while the outer 16 were used only to ensure accurate boundary conditions and their values were thrown out. The polar displacement for each atom was then calculated as the difference between its atom position and the mean position of the surrounding four opposite type cations. Defining the cross-section image to lie in the *x–z* Cartesian plane (*x*=[100], *z*=[001]) and all atoms on an *x*–*z* grid with A-sites at the integer positions and B-sites with 0.5,0.5 fractional coordinates, for an atom at grid position *i,j* this corresponds to mean neighbour positions (MNP) of:


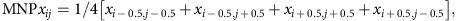



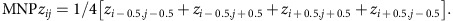


The polar displacement vector is defined with reversed order for A- and B-site centred atoms to maintain a consistent displacement vector sign (otherwise it would alternate directions):


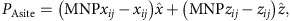



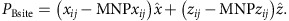


This convention defines a displacement vector direction nominally parallel to the full electrical polarization of the bulk crystal structure.

Determination of the data set pixel resolution is needed to accurately convert the measured polar displacement into informative distance units, that is, pm. The sample is fully coherent to the GdScO_3_ substrate so the in-plane lattice parameter of the film in the data set is equated to the nominal GdScO_3_ [110]_O_ (pseudocubic) lattice parameter of 3.9673 Å (ref. 44). For the distortion-corrected data set the resulting calculated pixel resolution was 30.09 pm per pixel.

### Molecular dynamics simulations

MD simulations of the compositionally graded Ba_*x*_Sr_1−*x*_TiO_3_ heterostructures were conducted with a 10 × 10 × *n* (*n*=36,144) perovskite-type supercell using a bond-valence-based interatomic potential^45^,^46^,^47^,^48^,^49^. The composition changes from BaTiO_3_ to Ba_0.6_Sr_0.4_TiO_3_ in steps (in each step, Ba concentration changes 5%) and back to BaTiO_3_ to satisfy periodic boundary conditions. The temperature is controlled by the Nosé–Hoover thermostat with a thermal inertia parameter *M*_*s*_=1.0 and the in-plane dimensions of each unit cell are fixed as 4 Å × 4 Å. The pressure in the out-of-plane direction is maintained at 0.1 MPa by the Parrinello–Rahman barostat^50^ implemented in LAMMPS.

### Topography and domain structure

Topographic study of the films was carried out using atomic force microscope (MFP-3D, Asylum Research). Dual AC Resonance Tracking piezoresponse force microscopy was used to image the domain structure.

### Electrical measurements

Characterization of electronic, ferroelectric and dielectric properties was completed for at least ten capacitors on multiple samples of each heterostructure variant. Measurements were completed on symmetric capacitor structures using SrRuO_3_ top and bottom electrodes^51^. The current–voltage characteristics and polarization–electric field hysteresis loops were measured using a Precision Multiferroic Tester (Radiant Technologies, Inc.). This ferroelectric tester employs the Virtual Ground method to measure ferroelectric polarization–electric field hysteresis loops. The room temperature low-field permittivity and the loss tangent were measured using an E4980A LCR metre (Agilent Technologies) was measured with a 10 kHz a.c. excitation voltage of 5 mV.

### Data availability

The data that support the findings of this study are available from the corresponding author upon request.

## Additional information

**How to cite this article:** Damodaran, A. R. *et al*. Large polarization gradients and temperature-stable responses in compositionally-graded ferroelectrics. *Nat. Commun.* **8**, 14961 doi: 10.1038/ncomms14961 (2017).

**Publisher's note:** Springer Nature remains neutral with regard to jurisdictional claims in published maps and institutional affiliations.

## Supplementary Material

Supplementary Information Supplementary Figures, Supplementary Notes and Supplementary References
