# Supplementary material for: Large polarization gradients and temperature-stable responses in compositionally-graded ferroelectrics
Source: Nat Commun. 2017 May 10;8:14961. doi: 10.1038/ncomms14961 (PMC5436105; doi:10.1038/ncomms14961)
Supplement: Supplementary Information — Supplementary Figures, Supplementary Notes and Supplementary References [file ncomms14961-s1.pdf]

**Supplementary Note 1: Heterostructure Design**

The current study requires the consideration of several potentially competing factors including strain, strain gradient/flexoelectric effects, depolarization, defects, effects from microstructural inhomogeneities (such as domain structures), etc.; all of which make a systematic study difficult. As such, we focus on $Ba_{1-x}Sr_xTiO_3$ heterostructures for a number of reasons. First, $Ba_{1-x}Sr_xTiO_3$-based ferroelectrics are good candidates for actual applications and are known for their high tunability, good radio-frequency loss characteristics, and have been found to be superior to other candidate materials for near-ambient radio-frequency/microwave applications[1-3]. Second, the lattice parameters, magnitude of ferroelectric polarization, ferroelectric domain structure, and various temperature-dependent phase transitions in the $Ba_{1-x}Sr_xTiO_3$ system are strongly dependent on both the Ba/Sr ratio[4-7] and epitaxial strain[8] and such features have been well studied in bulk and in uniform thin-film samples. Finally, because of this we can create model versions of these materials as (001)-oriented, single-crystal, and monodomain epitaxial films which provide for an ideal testbed for the study of compositional- and strain-gradient effects.

Specifically, we focus on uniform and compositionally-graded $Ba_{1-x}Sr_xTiO_3$ ($0 \leq x \leq 0.4$) heterostructures. The two end-members are: 1) $Ba_{0.6}Sr_{0.4}TiO_3$, which in the bulk exists as a cubic paraelectric (*Pm*3*m*) with a lattice parameter of $a$ = 3.965 Å at room temperature, and 2) $BaTiO_3$, which in the bulk exists as a tetragonal ferroelectric (*P*4*mm*, $T_C \approx 120°C$) at room temperature with lattice parameters $a$ = 3.992 Å and $c$ = 4.036 Å (pseudocubic lattice parameter, $a_{pc}$ = 4.006 Å)[4-7]. A compressive in-plane strain of 1% that occurs in coherently-strained $BaTiO_3$ films grown on $GdScO_3$ (110) substrates (pseudocubic lattice parameter, $a_{pc}$ = 3.967 Å) results in an out-of-plane tensile strain of 1.7% (with contributions from both Poisson and electrostriction effects) and is also

accompanied by enhancements of the ferroelectric $T_C$ by more than 400°C[8]. On the other hand, $Ba_{0.6}Sr_{0.4}TiO_3$ possesses negligible lattice mismatch (+0.05%) with the $GdScO_3$ (110) substrates and, consequently, $Ba_{0.6}Sr_{0.4}TiO_3$ films grown on $GdScO_3$ (110) substrates remains as a cubic paraelectric at room temperature. This is also seen in the temperature-dependent evolution of polarization predicted using Ginzburg-Landau-Devonshire-based models (Supplementary Figure 1a) for the two endmembers. The dielectric responses for strained-films of each of these end-members peak at their respective $T_C$ and demonstrate a strong temperature-dependence near the Curie point that is often undesirable for applications. In order to design for temperature-stable performance, in this work we focus on films where we gradually change the composition between the two end-member compositions across the film thickness. Since the phase transition characteristics of $Ba_xSr_{1-x}TiO_3$ films are a monotonic function of the Ba/Sr ratio, grading the composition across the thickness can result in a diffuse ferroelectric-to-paraelectric phase transition that extends between the $T_C$ of the two end-member compositions and thereby achieves enhanced, temperature-independent responses[9]. This also suggests that such compositional gradients, when used in conjunction with epitaxial strain, can result in an even broader temperature regime for temperature-insensitive dielectric responses as the $T_C$ of the end

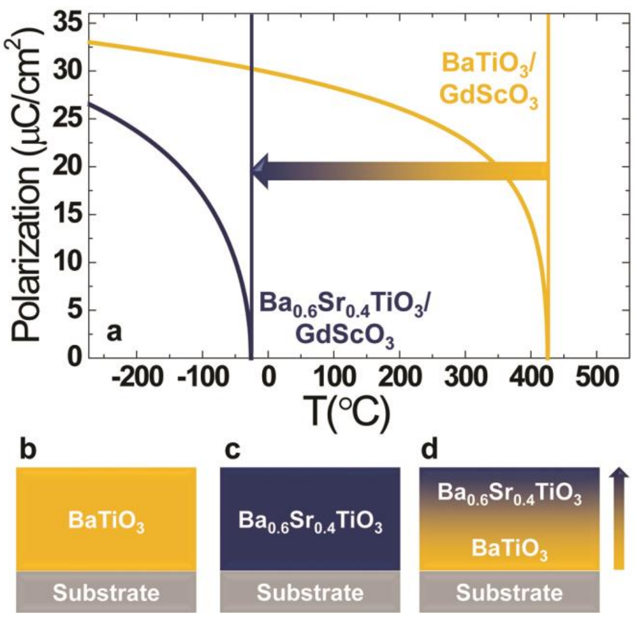


**Supplementary Figure 1. Rationale for heterostructure design. a,** Predicted temperature-evolution of polarization in coherently-strained $Ba_{0.6}Sr_{0.4}TiO_3/GdScO_3$ (110) and $BaTiO_3/GdScO_3$ (110) heterostructures. Schematic illustrations of the three sample variants studied in this work including **b,** single-layer $BaTiO_3$, **c,** single-layer $Ba_{0.6}Sr_{0.4}TiO_3$, and **d,** compositionally-graded heterostructures (which smoothly transition from $BaTiO_3$ to $Ba_{0.6}Sr_{0.4}TiO_3$ from the substrate to the film surface).

member compositions in the strained state are further apart. In order to investigate such effects, we employed pulsed-laser deposition to synthesize three different kinds of $Ba_xSr_{1-x}TiO_3$-based thin film heterostructures on $SrRuO_3$-buffered $GdScO_3$ (110) substrates: 1) single-layer $BaTiO_3$ (Supplementary Figure 1b), 2) single-layer $Ba_{0.6}Sr_{0.4}TiO_3$ (Supplementary Figure 1c), and 3) compositionally-graded heterostructures (which smoothly transition from $BaTiO_3$ to $Ba_{0.6}Sr_{0.4}TiO_3$ from the substrate to the film surface, Supplementary Figure 1d). We have included the end-member films of uniform composition in the study to enable a systematic analysis of the ferroelectric properties of compositionally-graded heterostructures in comparison with uniform composition films synthesized using the same growth conditions.

## Supplementary Note 2: Additional X-ray Diffraction Studies

For both the single-layer and compositionally-graded heterostructures studied herein, wide-angle $\theta$-$2\theta$ X-ray diffraction patterns were obtained with a Panalytical X'Pert Pro X-ray Diffraction (XRD) machine with a Cu source (Supplementary Figure 2). All heterostructures studied were single-phase, fully-epitaxial, and (001)-oriented. The presence of thickness fringes (Pendellösung fringes) about the central Bragg peak ($SL_{(0)}$ 002) of both uniform endmember-composition films, attest to the

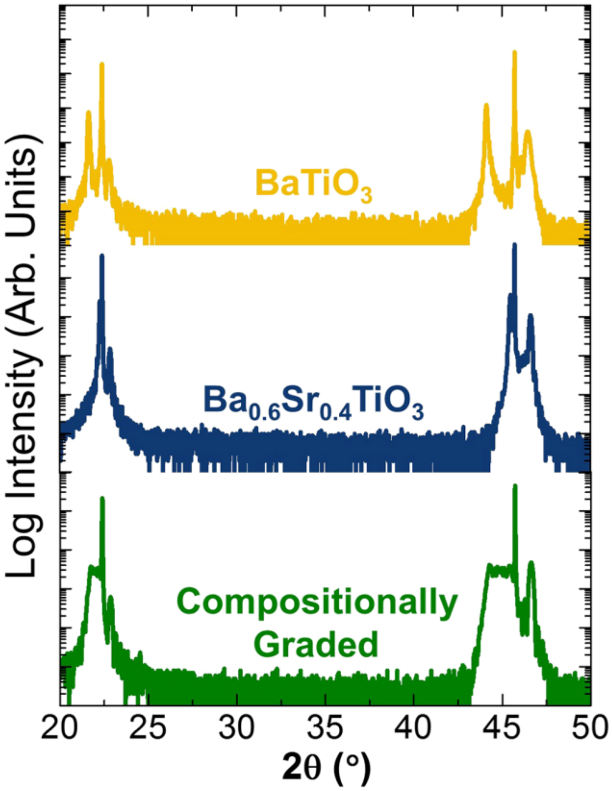


**Supplementary Figure 2. X-ray diffraction studies.** Wide-angle $\theta$-$2\theta$ scans for homogenous and compositionally-graded $Ba_{1-x}Sr_xTiO_3$ heterostructures indicating all films are single phase.

high crystallinity of the films, the precise nature of the interfaces, and the overall smoothness of the films studied. The same setup was used for off-axis X-ray reciprocal space mapping (RSM) studies about the 103- and 332-diffraction conditions of the compositionally-graded heterostructure and substrate, respectively, and reveal that the entire thickness of the heterostructure remains coherently strained to the substrate (main text Fig. 1b).

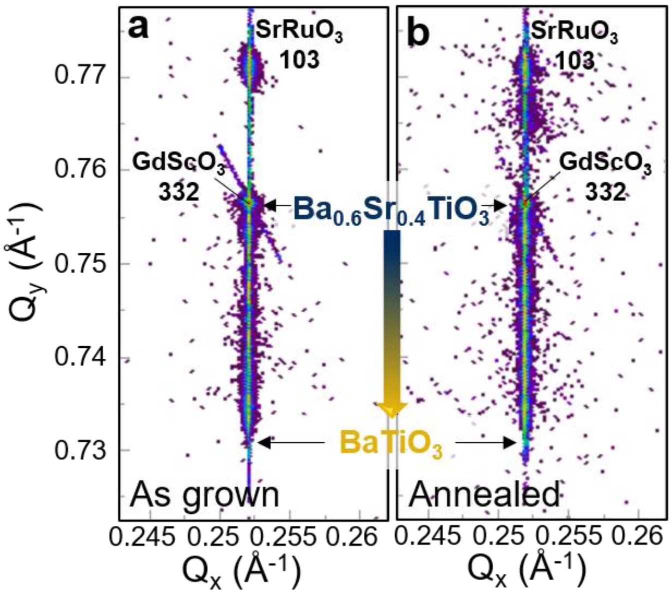


**Supplementary Figure 3. Stability of samples.** Off-axis RSMs for compositionally-graded $Ba_{1-x}Sr_xTiO_3$ films showing lattice parameters extending of a large angular range for **a,** as-grown and **b,** 50 hour, 500°C annealed heterostructures.

We also present X-ray RSM studies on the compositionally-graded heterostructures as a measure of the stability of samples after robust annealing procedures. In the main text, we provide ample proof of the presence of a compositional-gradient (both from X-ray diffraction studies, RSMs, RBS, and TEM, main text Figs. 1a-e). We conducted numerous high-temperature anneals and studies following thermal cycling of the various heterostructures and in all cases the films remain (to the best of experimental understanding) unchanged following those thermal excursions. To demonstrate this stability we provide an RSM of the as-grown heterostructure (Supplementary Figure 3a) and that for the same heterostructure after an aggressive annealing procedure – 50 hours at 500°C in air (Supplementary Figure 3b). What is clear from the study is that the compositional gradient remains intact – as evidenced by the continuously varying lattice parameter in the $Q_y$ direction for the films which is essentially unchanged. We also note that other indicators – such as

dielectric response – confirm this observation that repeated or long-term exposure to high-temperatures does not impact the gradient structure.

## Supplementary Note 3: Low- and High-Resolution STEM Images

Here we provide cross-section STEM studies at different resolutions (Supplementary Figure 4a, b). Low-resolution images (main text Fig. 1d and Supplementary Figure 4a) reveal no ferroelastic domains, extended defects such as dislocations, etc. High-resolution STEM images reveal sharp interfaces between the compositionally-graded film and the bottom electrode (Supplementary Figure 4b), and also attests to the continuous nature of the lattice at unit-cell resolution (main text Fig. 2a). These studies reveal the high-quality, single-crystal nature of the compositionally-graded heterostructures without any significant microstructural inhomogeneity, and as such, they represent a model test structure to explore the role of compositional and strain gradients on the evolution of polarization and properties.

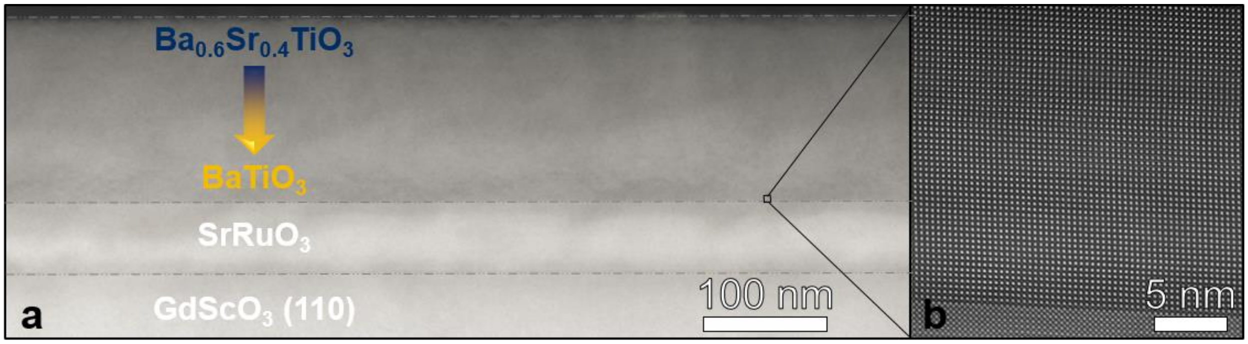


**Supplementary Figure 4. Scanning transmission electron microscopy studies. a,** Low-resolution STEM image showing the absence of any microstructural variations including grains, domains, dislocations, etc. **b,** High-resolution STEM image near the film/bottom-electrode interface revealing atomically-sharp interfaces and the single crystal, epitaxial nature of the compositionally-graded heterostructures.

## Supplementary Note 4: Nanobeam Electron Diffraction (NBED)

Strain mapping using NBED is a technique that calculates elastic strains and rigid-body crystal rotations directly from NBED patterns[10]. In STEM, an electron probe with nanoscale

diameter is formed with a small ~2 mrad convergence semi-angle, and a set of diffraction patterns are collected in a grid pattern at each beam position on the sample. Each NBED pattern contains a local measurement of the crystal orientation and elastic strain. The lattice spacing and angles can be extracted from the diffraction spots by calculating the position of their centroids, and the diffractions spots in an unstrained pattern are used as an internal calibration to determine the diffraction pixel size (as depicted in Supplementary Figure 5). Unlike other TEM-based strain mapping techniques such as geometric phase analysis (GPA), NBED has relatively few limitations in terms of sample, scan geometry, and mapping area. For example, NBED has been used to map out elastic strains in a GaAs/GaAsP multilayer device over a 1x1 μm region containing significant internal crystal rotations while still maintaining an elastic strain resolution of 0.1%[10,11].

In the present experiment, patterns were collected in a rectangular 64 × 256 pixel grid with 2.5 nm spacing between probes. The probe size was approximately 1 nm (smaller than the probe spacing), giving a spatial resolution of the NBED measurements equal to the probe spacing. All

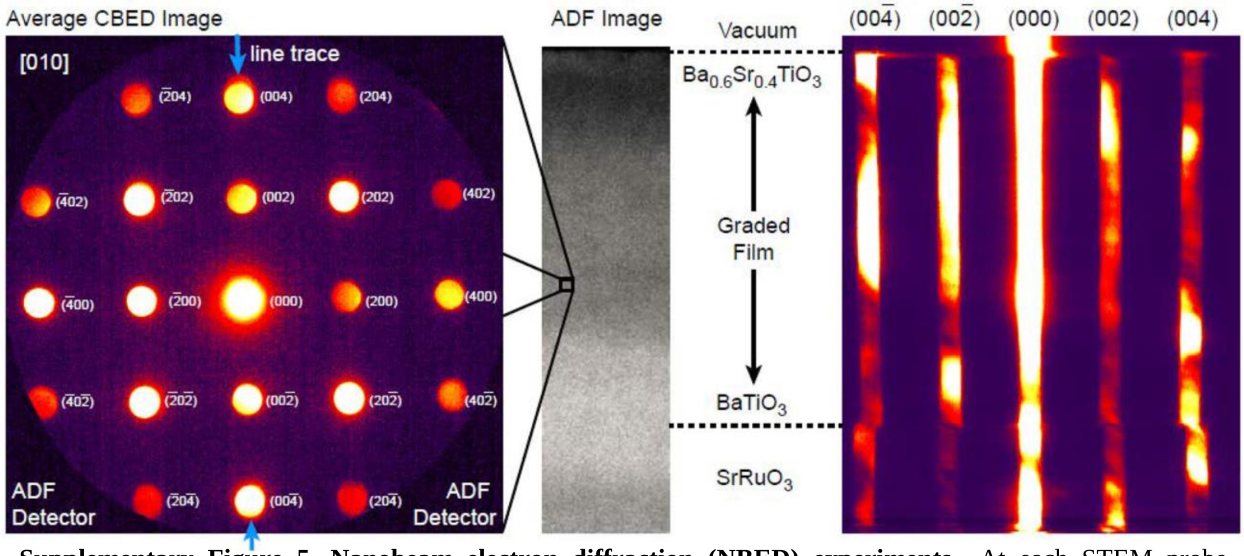


**Supplementary Figure 5. Nanobeam electron diffraction (NBED) experiments.** At each STEM probe position, a full image of the diffraction-space convergence beam electron diffraction (CBED) pattern was recorded. (left) Average of all CBED images from the compositionally-graded heterostructures, recorded along the [020] zone axis. The annular dark-field (ADF) detector is visible as a circular aperture at the edges. (center) Signal recorded by the ADF detector. (right) Line trace which is 100 pixels wide from the mean CBED images as a function of depth along the film growth axis, position shown as blue arrows on average CBED image. The abrupt change in *c*-axis disk spacing is clearly visible between the $SrRuO_3$ and $BaTiO_3$ layers.

patterns were converted to edge measurements by convolution with a Sobel kernel, and then the diffracted disk positions were measured by using phase correlation (with sub-pixel precision) between the center (unscattered) disk and the entire pattern[10]. This procedure suppresses fitting errors due to patterns inside each disc from dynamical diffraction effects. A sub-pixel resolution cross-correlation algorithm was applied to detect the centroids of the NBED disks. The average pattern from the substrate was selected as the reference to calibrate all other diffraction patterns ($g_i^{ref}$). Subsequently, for each diffraction pattern the transformation matrix $T$ was computed from the diffraction vectors $g_i$ using $g_i = T\ g_i^{ref}$. Polar decomposition was used to separate the transformation matrix $T$ into a rotation matrix $R$ and a symmetric strain matrix $U$. In-plane and out-of-plane strains were computed as $\varepsilon_{xx} = 1\text{-}U_{00}$ and $\varepsilon_{yy} = 1\text{-}U_{11}$ and the shear and rotation can be computed using $\varepsilon_{xy} = -\ U_{01}$ and $\theta = sin^{-1}R_{01}$. Note that $\varepsilon_{xx}$, $\varepsilon_{yy}$, and $\varepsilon_{xy}$ have negative signs because the strain measurements take place in reciprocal space. Following extraction of the local strain, averaging was completed by taking the mean of all measurements perpendicular to the growth direction.

## Supplementary Note 5: Current-Voltage Characteristics

A comparative study of the current density – electric field leakage characteristics (Supplementary Figure 6) for the compositionally-graded heterostructure with devices based on uniform end-member composition of $BaTiO_3$ and $Ba_{0.6}Sr_{0.4}TiO_3$

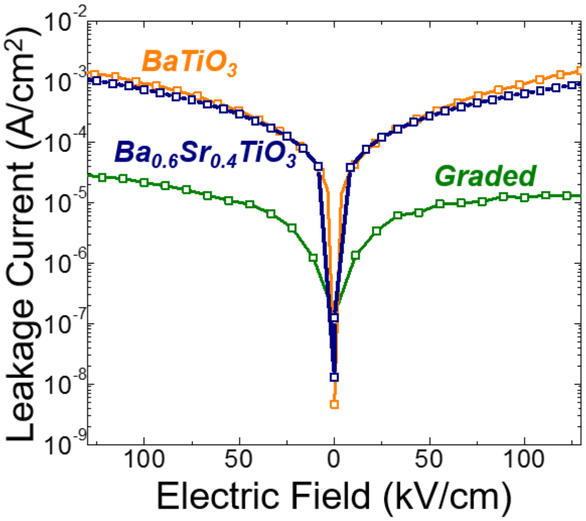


**Supplementary Figure 6. Current-electric field leakage characteristics.** Substantially reduced leakage in compositionally-graded heterostructure devices as compared to $BaTi0_3$ and $Ba_{0.6}Ti_{0.4}O_3$

heterostructures reveals a resistivity of ~$10^{10}$ Ω-cm. Such high values of resistivity that are nearly two orders of magnitude greater than that measured for the $BaTiO_3$- and $Ba_{0.6}Sr_{0.4}TiO_3$-based heterostructure devices, and are comparable to values measured for bulk single crystals of $BaTiO_3$[12].

## Supplementary Note 6: Molecular Dynamics (MD) Simulations for the $Ba_xSr_{1-x}TiO_3$ Heterostructures

MD simulations of the compositionally-graded $Ba_xSr_{1-x}TiO_3$ heterostructures were conducted with a $10\times10\times n$ ($n$ = 36,144) perovskite-type supercell using a bond-valence-based interatomic potential[13-17]. The composition changes from $BaTiO_3$ to $Ba_{0.6}Sr_{0.4}TiO_3$ in steps (in each

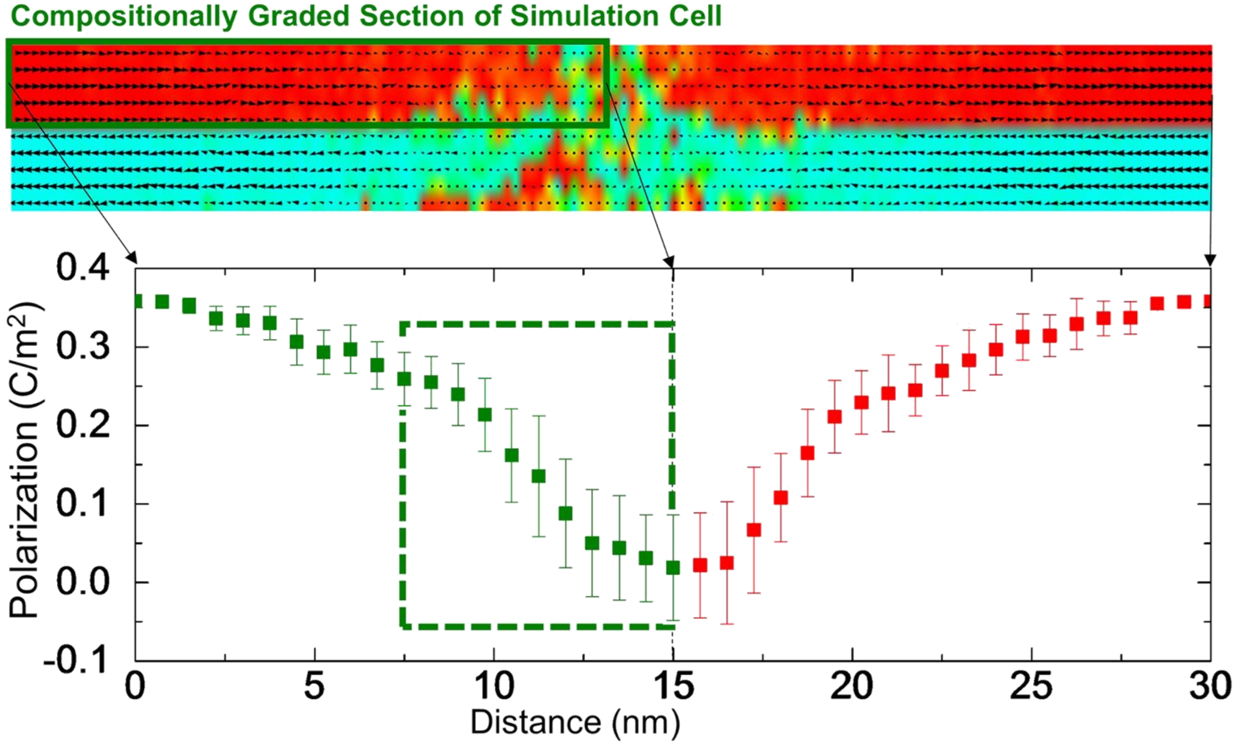


**Supplementary Figure 7. Molecular dynamics simulations.** Polarization profile for a compositionally-graded heterostructure (30 nm). A large polarization gradient forms in the compositionally-graded $Ba_xSr_{1-x}TiO_3$. In order to compensate the depolarization field induced by the polarization gradient, a 180° domain wall pattern is observed in the simulations. We focus on the segment with polarization along the [001] (surrounded by the green square) to simulate the experimental set up. The other domain compensates the depolarization field in the simulation, similar to the role of space charges in the experimentally-grown, compositionally-graded heterostructures.

step, Ba concentration changes 5%) and back to $BaTiO_3$ to satisfy the periodic boundary conditions. Also, due to the periodicity of the supercell going from $BaTiO_3$ to $Ba_{0.6}Sr_{0.4}TiO_3$ and back to $BaTiO_3$, there is no potential drop through the supercell. In the MD simulation, a 180° domain pattern forms as the non-uniform polarization in only one direction will cause a depolarization field and make the system unstable. In this study, we only investigate the part of the supercell with polarization in one direction (Supplementary Figure 7). This part corresponds to the experimental set up and the other domain works as a compensation of the depolarization field, similar to the role of screening charges as elaborated upon in the main text.

The temperature is controlled by the Nosé-Hoover thermostat with a thermal inertia parameter $M_s = 1.0$ and the in-plane dimension of each unit cell is fixed as 4 Å×4 Å. The pressure in the out-of-plane direction is maintained at 0.1 MPa by the Parrinello-Rahman barostat[18] implemented in LAMMPS. The bond-valence-based interatomic potential is parameterized from density functional theory calculations and can reproduce temperature-driven phase transitions of $BaTiO_3$ accurately. The instantaneous local polarization, $\mathbf{P}_\mathrm{u}(t)$, for each unit cell is calculated with $\boldsymbol{P_u}(t) = \frac{1}{V_u}\left(\frac{1}{8}\boldsymbol{Z}^*_{\mathrm{Ba}}\sum_{i=1}^{8}\boldsymbol{r}_{\mathrm{Ba},i}(t) + \boldsymbol{Z}^*_{\mathrm{Ti}}\boldsymbol{r}_{\mathrm{Ti}}(t) + \frac{1}{2}\boldsymbol{Z}^*_{\mathrm{O}}\sum_{i=1}^{6}\boldsymbol{r}_{\mathrm{O},i}(t)\right)$, where $V_u$ is the volume of a unit cell, $\boldsymbol{Z}^*_{\mathrm{Ba}}$, $\boldsymbol{Z}^*_{\mathrm{Ti}}$, and $\boldsymbol{Z}^*_{\mathrm{O}}$ are the Born effective charges of Ba, Ti, and O atoms[19], and $\boldsymbol{r}_{\mathrm{Ba},i}(t)$, $\boldsymbol{r}_{\mathrm{Ti},i}(t)$, and $\boldsymbol{r}_{\mathrm{O},i}(t)$ are the instantaneous atomic positions of Ba, Ti, and O atoms in a unit cell obtained from MD simulations. The dielectric permittivity $\varepsilon$ of a specific composition is calculated by[20]:

$$\langle \delta P_i^2 \rangle = \frac{k_B T \varepsilon_i}{V},$$

where $k_B$ is the Boltzmann constant, $T$ is the temperature, and $V$ is the characteristic volume in which polarization amplitude is spatially correlated. $\delta P_i$ is the polarization fluctuation of the $i$th

composition and defined as the difference of the polarization at time $t$, $P_i(t)$, and that at time 0, $P_i(0)$. A specific component of the dielectric permittivity is calculated from the same component of the polarization fluctuation. $\delta P_i^2$ is calculated in a time range of 10 ps. $V$ is set as 512 $Å^3$(≈8 unit cell), according to the reference[17].

### Supplementary Note 7: Topography and Domain Structure Using Piezoresponse Force Microscopy

Here, for reference, we provide Dual AC Resonance Tracking (DART) piezoresponse force microscopy (PFM) images of a 150 nm thick, compositionally-graded heterostructure revealing a smooth topography with a roughness of less than 500 pm (Supplementary Figure 8a). The uniform contrast in both the out-of-plane (vertical PFM, Supplementary Figure 8b) and in-plane (lateral PFM, Supplementary Figure 8c) suggests the absence of any 180° domain patterns and is indicative of the fact that the films are monodomain in nature.

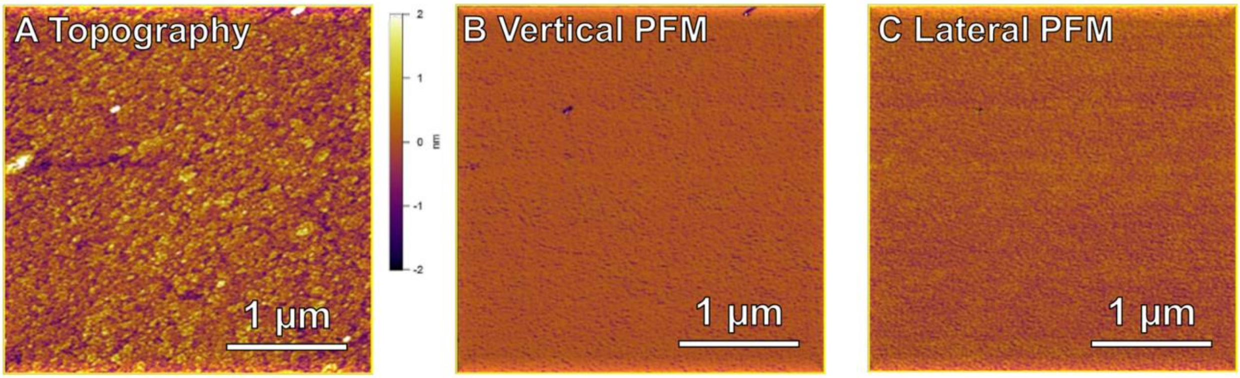


**Supplementary Figure 8. Scanning-probe microscopy-based studies. a,** Topography, **b,** vertical PFM, and **c,** lateral PFM images for compositionally-graded heterostructure. Lack of any contrast in the PFM images suggests a monodomain film.

## Supplementary Note 8: Proposed Compensation Mechanism

The nanoscale polarization mapping studies (main text Fig. 2b) reveal that the as-grown, compositionally-graded films going from $BaTiO_3$ at the bottom to $Ba_{0.6}Sr_{0.4}TiO_3$ at the surface exhibit both a polarization gradient wherein the polarization reduces from the bottom to the top of the film and also a preferred direction of polarization which points from bottom to the top of the film (Supplementary Figure 9). Under this scenario, a net negative space charge ($-\sigma_{sc}$) is needed to compensate the polarization gradient. The term space charge is a rather generic term that is used to describe regions in space that are electrically charged, and can arise either from a depletion or accumulation of mobile charge carriers. $Ba_xSr_{1-x}TiO_3$–based films grown under oxidizing conditions are typically p-type semiconductors (due to barium vacancies and titanium-oxygen divacancies under Ti- and Ba-rich conditions, respectively)[21]. For such systems, negative space charges can only be generated via a depletion of the majority hole carriers which leave behind and electrically-charged depletion region of immobile/fixed negatively-charged ions. This is what sets the direction of preferred polarization for the compositionally-graded heterostructures, and in turn the direction of the shift in hysteresis loops of the as-grown devices. In turn, such a depletion of holes from within the compositionally-graded heterostructure not only allows the stabilization of large polarization gradients, but also minimizes leakage or conduction from the

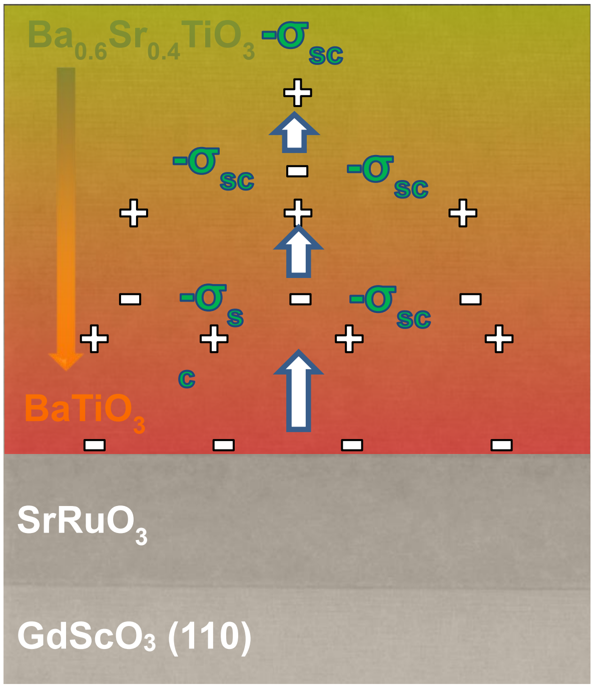


**Supplementary Figure 9. Mechanism for hysteresis-offsets.** Schematic of polarization gradient in as-grown films showing nature of space charge compensation required to stabilize said gradient.

drift of majority carriers (as evidenced by the reduction in the current density compared to the uniform, end-member compositions, Supplementary Figure 6). In summary, the shift in hysteresis loops arises the nature of polarization-gradient screening facilitated by the defect-structures and the related p-type semiconductivity of the compositionally-graded heterostructures. If the compositionally-graded heterostructures were n-type, an equal but opposite shift is expected.

**Supplementary Note 9: Ferroelectric Measurements**

Detailed characterization of ferroelectric properties was completed for at least 10 capacitors on multiple samples of each heterostructure variant. Measurements were completed on symmetric capacitor structures using $SrRuO_3$ top and bottom electrodes. Detailed polarization-electric field hysteresis studies were completed for the uniform-composition and compositionally-graded heterostructures that reveal well-centered loops for the uniform composition films, but an offset of ~25 kV $cm^{-1}$ for the compositionally-graded heterostructures (Supplementary Figure 10a). The observed shift is indicative of a built-in potential which can arise from flexoelectric effects[22-25] as well as space-charge distributions[26] in the compositionally-graded heterostructures. Field-cooling experiments were conducted to understand the relative importance of the extrinsic space charge and intrinsic flexoelectric driving forces for such shifted loops. The compositionally-graded heterostructure devices were heated to 500°C and subsequently cooled under applied electric fields of $\pm$100 kV $cm^{-1}$. The ability to completely reverse the hysteresis offsets in these field-cooling

experiments (Supplementary Figure 10b) highlight the dominant contribution from extrinsic charged defect-based mechanisms.

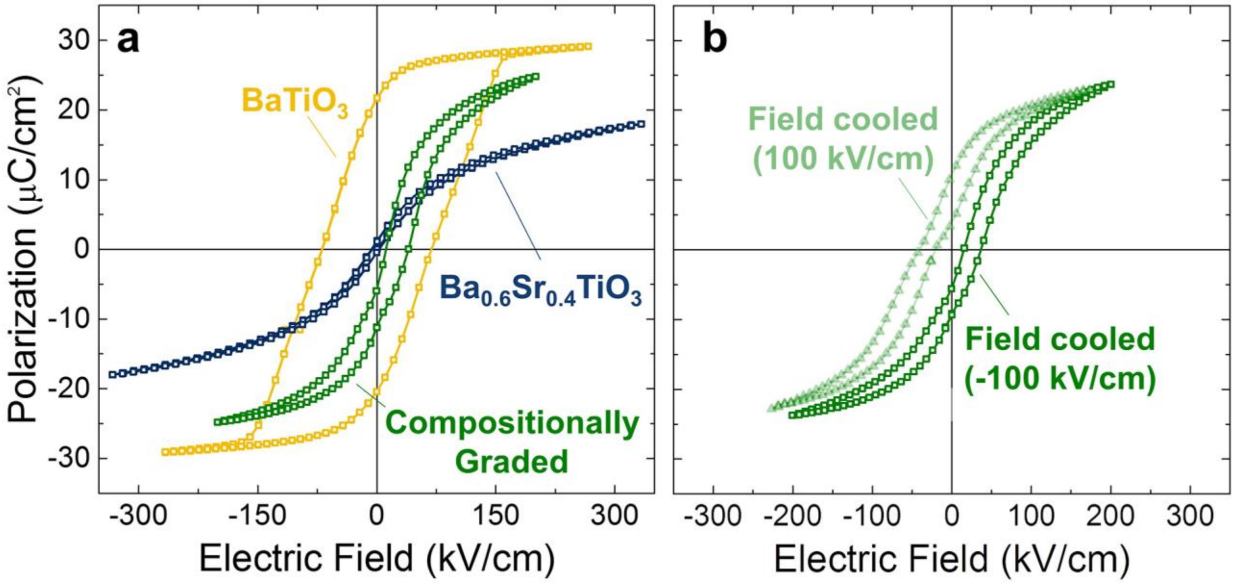


**Supplementary Figure 10. Polarization-electric field hysteresis loops.** Measured at 10 kHz for uniform-composition **a,** $BaTiO_3$ (yellow), $Ba_{0.6}Sr_{0.4}TiO_3$ (blue) and compositionally-graded (green) heterostructures in the as-grown state, and **b,** after field-cooling for the compositionally-graded heterostructures.

## Supplementary References


1. Subramanyam, G. *et al*. Challenges and opportunities for multi-functional oxide thin films for voltage tunable radio frequency/microwave components. *J. Appl. Phys.* **114**, 191301 (2013).
2. Tagantsev, A. K., Sherman, V. O., Astafiev, K. F., Venkatesh, J. & Setter, N. Ferroelectric materials for microwave tunable applications. *J. Electrocer.* **11**, 5–66 (2003).
3. York, R. A. "Tunable Dielectrics for RF Circuits". *Multifunctional Adaptive Microwave Circuits And Systems*. M. Steer and W. D. Palmer. SciTech Publishing, 2009.
4. Lines, M. E. & Glass, A. M. in *Principles and Applications of Ferroelectrics and Related Materials* (Oxford University Press, Oxford, 2001).
5. Strukov, B.A., Levanyuk, A. P. in *Ferroelectric Phenomena in Crystals* (Springer-Verlag Berlin Heidelberg, 1998).
6. Remmel, T., Gregory, R. & Baumert, B. Characterization of barium strontium titanate films using XRD. *JCPDS-International Centre for Diffraction Data* **41**, 38 (1999).
7. Basmajian, J. A. & DeVries, R. C. Phase Equilibria in the System $BaTiO_3$–$SrTiO_3$. *J. Am. Ceram. Soc.* **40**, 373-376 (1957).
8. Choi, K. J. *et al*. Enhancement of Ferroelectricity in Strained $BaTiO_3$ Thin Films. *Science* **306**, 1005-1009 (2004).
9. Shut, V. N., Syrtsov, S. R., Trublovsky, V. L. & Strukov, B. A. Compositionally graded ceramics based on $Ba_{1-x}Sr_xTiO_3$ solid solutions. *Inorg. Mater.* **47**, 87-92 (2011).
10. Ozdol, V. B. *et al*. Strain mapping at nanometer resolution using advanced nano-beam electron diffraction. *Appl. Phys. Lett.* **106**, 253107 (2015).
11. Hÿtch, M. J., Snoeck, E. & Kilaas, R. Quantitative measurement of displacement and strain fields from HREM micrographs. *Ultramicroscopy* **74**, 131-146 (1998).
12. Boyeaux, J. P. & Michel-Calendini, F. M. Small polaron interpretation of $BaTiO_3$ transport properties from drift mobility measurements. *J. Phys. C: Solid State Phys.* **12**, 545 (1979).
13. Liu, S., Grinberg, I. & Rappe, A. M. Development of a bond-valence based interatomic potential for $BiFeO_3$ for accurate molecular dynamics simulations. *J. Phys. Condens. Matter* **25**, 102202 (2013).
14. Liu, S., Grinberg, I. & Rappe, A. M. Exploration of the intrinsic inertial response of ferroelectric domain walls via molecular dynamics simulations. *Appl. Phys. Lett.* **103**, 232907 (2013).
15. Shin, Y. -H., Son, J. -Y., Lee, B. -J., Grinberg, I. & Rappe, A. M. Order-disorder character of $PbTiO_3$. *J. Phys. Condens. Matter* **20**, 015224 (2008).

16. Shin, Y. -H., Cooper, V. R., Grinberg, I. & Rappe, A. M. Development of a bond-valence molecular-dynamics model for complex oxides. *Phys. Rev. B* **71**, 054104 (2005).

17. Grinberg, I., Cooper, V. R. & Rappe, A. M. Relationship between local structure and phase transitions of a disordered solid solution. *Nature* **419**, 909-911 (2002).

18. Parrinello, M. & Rahman, A. Crystal Structure and Pair Potentials: A Molecular-Dynamics Study. *Phys. Rev. Lett.* **45**, 1196-1199 (1980).

19. Ghosez, Ph., Michenaud, J. -P. & Gonze, X. Dynamical atomic charges: The case of $ABO_3$ compounds. *Phys. Rev. B* **58**, 6224-6240 (1998).

20. Wemple, S. H., DiDomenico, M. & Jayaraman, A. Electron Scattering in Perovskite-Oxide Ferroelectric Semiconductors. *Phys. Rev.* **180**, 547-556 (1969).

21. Erhart, P. & Albe, K. Modeling the electrical conductivity in $BaTiO_3$ on the basis of first-principles calculations. *Journal of Applied Physics* **104**, 044315 (2008).

22. Zubko, P., Catalan, G. & Tagantsev, A. K. Flexoelectric Effect in Solids. *Annu. Rev. Mater. Res.* **43**, 387-421 (2013).

23. Yudin, P. V., Tagantsev, A. K. & Tagantsev, A. K. Fundamentals of flexoelectricity in solids. *Nanotechnology* **24**, 432001 (2013).

24. Marvan, M., Chvosta, P. & Fousek, J. Theory of compositionally graded ferroelectrics and pyroelectricity. *Appl. Phys. Lett.* **86**, 221922 (2005).

25. Sai, N., Meyer, B. & Vanderbilt, D. Compositional Inversion Symmetry Breaking in Ferroelectric Perovskites. *Phys. Rev. Lett.* **84**, 5636-5639 (2000).

26. Okatan, M. B., Mantese, J. V. & Alpay, S. P. Effect of space charge on the polarization hysteresis characteristics of monolithic and compositionally graded ferroelectrics. *Acta Mater.* **58**, 39-48 (2010).
